# Supplementary material for: Enhanced triplet superconductivity in next-generation ultraclean UTe2
Source: Proc Natl Acad Sci U S A. 2024 Sep 6;121(37):e2403067121. doi: 10.1073/pnas.2403067121 (PMC11406238; doi:10.1073/pnas.2403067121)
Supplement: Supplementary file 1 — Appendix 01 (PDF) [file pnas.2403067121.sapp.pdf]

PNAS

Supporting Information for

Enhanced triplet superconductivity in next generation ultraclean UTe_2

Z. Wu, T. I. Weinberger, J. Chen, A. Cabala, D. V. Chichinadze, D. Shaffer, J. Pospisil, J. Prokleska, T. Haidamak, G. Bastien, V. Sechovsky, A. J. Hickey, M. J. Mancera-Ugarte, S. Benjamin, D. E. Graf, Y. Skourski, G. G. Lonzarich, M. Valiska, F. M. Grosche, A. G. Eaton

Corresponding author: A. G. Eaton
E-mail: alex.eaton@phy.cam.ac.uk

This PDF file includes:

Supporting text
Figs. S1 to S9
SI References

Z. Wu, T. I. Weinberger, J. Chen, A. Cabala, D. V. Chichinadze, D. Shaffer, J. Pospisil, J. Prokleska, T. Haidamak, G. Bastien, V. Sechovsky, A. J. Hickey, M. J. Mancera-Ugarte, S. Benjamin, D. E. Graf, Y. Skourski, G. G. Lonzarich, M. Valiska, F. M. Grosche, A. G. Eaton

Supporting Information Text

Sample preparation and characterization

In this section we compare characterization measurements of the sample measured by the PDO technique to high fields, for which the data are presented in Fig. 3 of the main text. We measured the superconducting transition of this sample by three different methods: (*A*) PDO, (*B*) superconducting quantum interference device (dc SQUID), and (*C*) specific heat. PDO was measured by connecting the same coil as was later used in the 70 T pulsed magnet onto a homemade low temperature probe by a coaxial cable. This was then measured on cooling to the base temperature (1.8 K) of a PPMS system at 0.02 K/min in zero applied field. The dc magnetic moment, M, was measured by a QD Magnetic Property Measurement System (MPMS). The curve shown in Fig. S1(*B*) was measured on warming with a 10 Oe field applied after a zero-field cool-down. Heat capacity (C_p) was measured by a standard QD PPMS heat capacity module.

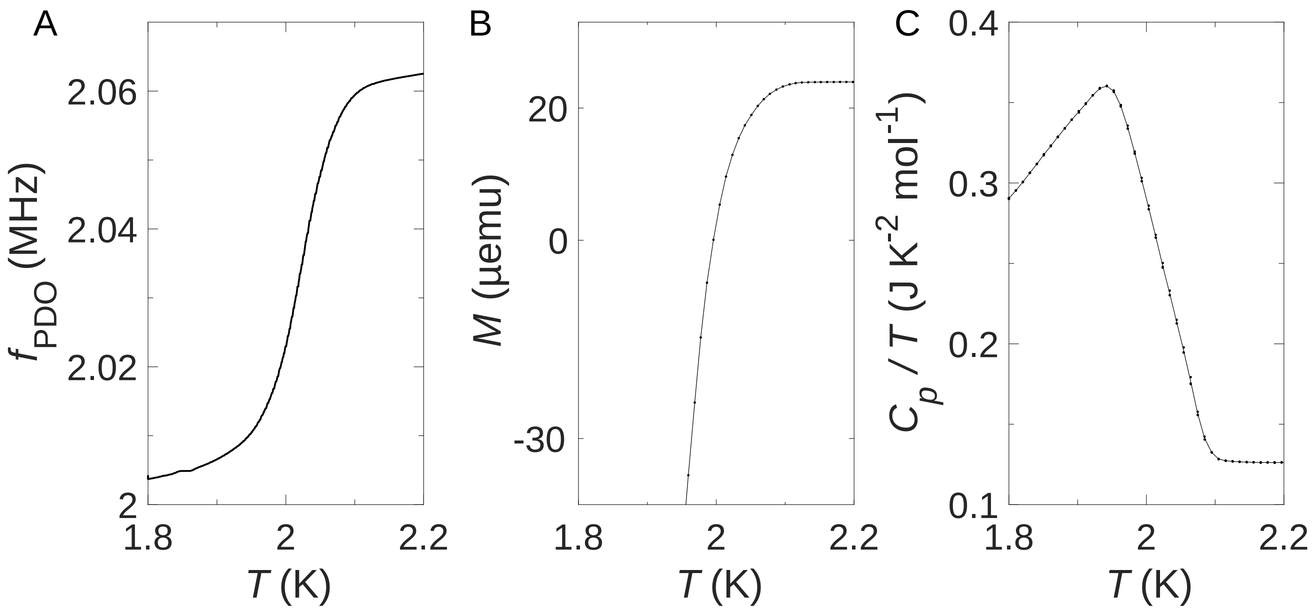

Fig. S1. Superconducting transition of the sample measured to high fields in Fig. 3 of the main text, measured at ambient magnetic field. The superconducting transition was measured by (*A*) PDO, (*B*) SQUID, and (*C*) heat capacity.

Figure S2 shows contacted and contactless resistivity measurements performed simultaneously on the same sample. A Gaussian is fitted to the derivative, with dashed lines marking the location of the Gaussian midpoint, 0.5σ, and 1σ. We find in Fig. 1 that very good correspondence between PDO and contacted resistivity measurements is observed by empirically taking the Gaussian centre of the derivative of the PDO signal plus 0.5σ. The PDO error bars in Fig. 1B are each of length 1σ (to represent an approximate uncertainty of $\pm$ 0.5σ).

Crystallographic orientation was calibrated by Laue diffraction as shown in Fig. S3. For pulsed field measurements, the data at 20° and 33° shown in Fig. 5 were obtained by mounting the sample on wedges of PEEK machined to the desired angles. The rotation study in dc magnetic fields presented in Fig. 4 was performed with a single-axis rotation probe utilizing a gear mechanism, with the rotation angle calibrated using a Hall sensor.

Phase mapping of UTe_2. All contacted resistivity measurements to determine the upper critical field (H_{c2}) of the SC1 phase, presented as solid diamonds in Fig. 1(*B*), were obtained on the RRR = 406 sample from Table 1 of the main text. This sample was oriented by Laue diffractometry and then securely mounted on a G10 sample board to enable easy orientation along each crystallographic axis. Figure S4 shows the raw data from which Fig. 1(b) is partly constructed. These data were obtained using the dc electrical transport module of a QD PPMS down to a base temperature of 0.5 K. Each data point was obtained by stabilizing the temperature and averaging over several measurements. $T_c(H)$ was defined by zero resistivity, which we identify as the first measurement point to fall below 0.1 μΩ cm on cooling. The excitation current for measurements with field applied along the a- and c-axes was 100 μA; the excitation current for measurements with field applied along the b-axis was 200 μA. Small applied currents were required to maintain the temperature stability, due to low cooling power for $T \lessapprox 1$ K.

Figure S5 shows the PDO signal for rising and falling magnetic field over the duration of a pulsed field measurement. Due to the high $\partial H/\partial t$ of a pulsed magnet, some amount of heating (from eddy currents and vortex motion) is inevitable.[2,3,4] On inspecting the up- and down-sweeps in Fig. S5, the location of the kink feature – which identifies the transition from SC1 to SC2 – has clearly moved to lower field on the down-sweep. This is highly likely to be an effect of heating during the pulse. Therefore, in Fig. 3 we use only the up-sweep data of each PDO measurement, to mitigate this effect.

Magnetization measurements to determine the lower critical field (H_{c1}) were obtained using the helium-3 option of a QD MPMS, for which the data are presented in Figure. S6. The sample was mounted inside a Kapton tube, with the field aligned along the a-axis. For each isothermal field sweep, the sample was first warmed up above its critical temperature and the magnet was turned off at high temperature. Then

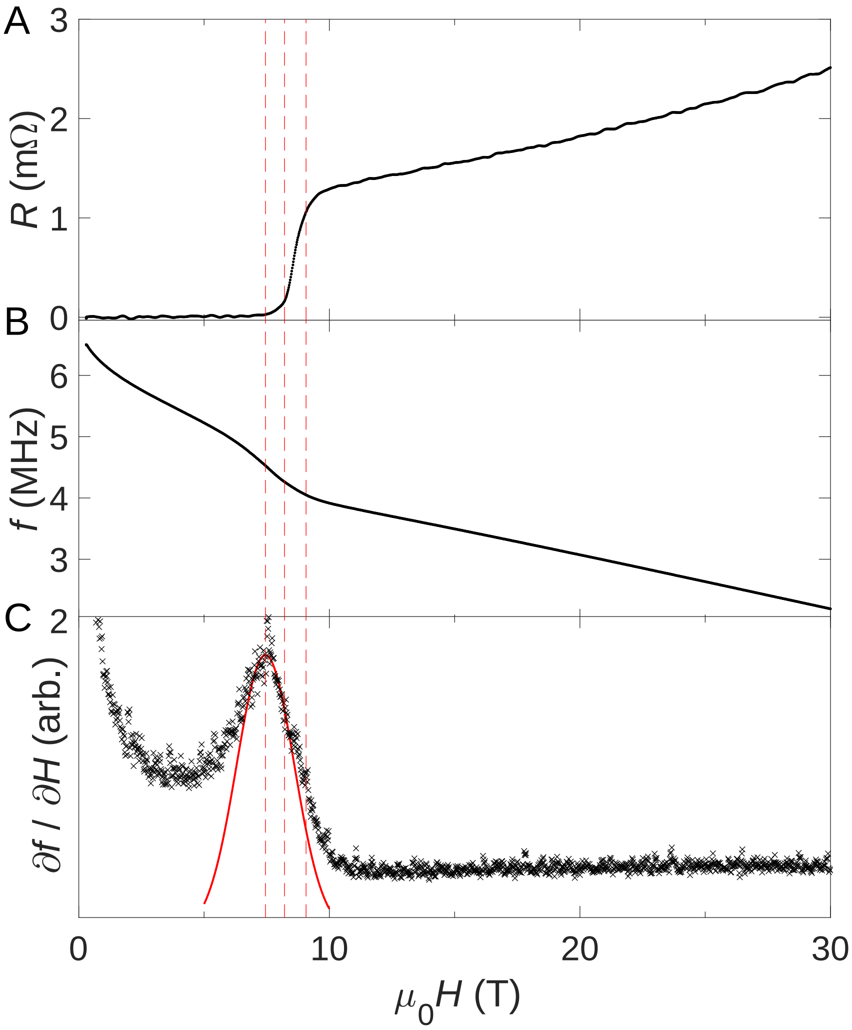

Fig. S2. Simultaneous measurement of (*A*) contacted resistivity and (*B*) contactless resistivity performed on the same sample. The derivative of the contactless resistivity data is given in (*C*).

the sample was cooled down to the assigned temperature in zero field. dc magnetic moment measurements were then performed with stabilized magnetic field.

When a sample is in the Meissner phase, it will be in a diamagnetic state of constant susceptibility.[7] In terms of moment versus field, a straight line is thus expected within the Meissner state. The lower critical field may therefore be identified as the lowest field value where the M vs H curve deviates from linearity (with a correction for the demagnetization effect, as detailed in e.g. refs.[8,9]). We fit a linear function to the data below 5 Oe at each temperature, which is then subtracted from each curve. The background-subtracted data for each temperature are shown in Fig. S6(iB). The flux penetration field H_p is then extracted by finding the first point that deviates from the flat line at each temperature. Following the discussion in ref.,[10] H_{c1} may be related to H_p via the expression:

$$H_{c1} = \frac{H_p}{\tanh\sqrt{0.36t/w}}, \qquad [1]$$

where t is the sample thickness and w is the sample width. For this measurement, with $H \parallel a$, t = 3.46 mm (along the a direction) and w = 0.51 mm.

We find that H_{c1} is enhanced for this new generation of higher quality samples (Fig. S6(*C*)), similar to the higher H_{c2} values shown in Fig. 1. We note that the H_{c1} value of ≈ 20 Oe we observe for $H \parallel a$ agrees well with a recent report of a similar study on MSF-grown UTe_2.[11]

Figure 3 shows the temperature evolution of the MM transition up to 3 K, over which interval it displays little change. We also tracked the evolution of H_m to higher temperatures, as shown in Figure S7. Whereas at low temperature H_m is very clearly visible by the sudden increase of the resistivity, at high temperatures this feature rounds out into a broad maximum, indicated with markers in Fig. S7(*A*).

In Fig. S7(*B*) we compare the temperature evolution of the MM transition between our study on a MSF sample with that reported previously in ref.[6] for a CVT specimen. Note that our study and that of ref.[6] are performed at different angles, so the location of H_m (at equivalent temperature) is slightly different. However, as we show in Figs. 5 & 6 the angular evolution of H_m is the same for MSF and CVT samples.

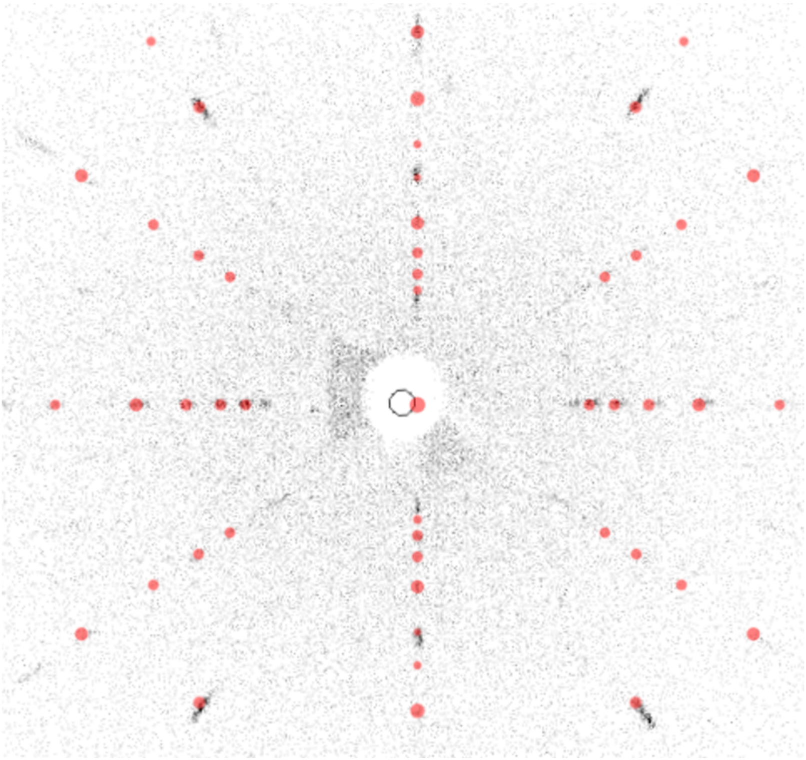

Fig. S3. Lauegram of a UTe_2 sample (black and white), overlaid with the expected diffraction pattern for x-rays incident on the (001) plane (in red). Single crystallinity is evidenced, with the [001] direction aligned to within 1°.

Furthermore, from the comparison in Fig. S7*B* it is clear that H_m displays a very similar temperature dependence for both types of sample. This indicates that the energy scale of the MM transition is unchanged between the two types of samples. We note that the MM transition at H_m is still observed even in very low quality samples that do not show SC1 superconductivity down to temperatures ≈ 0.5 K.[13] Given that we observe no change in the profile of this transition for this new generation of ultraclean crystals, we conclude that the MM transition is an intrinsic feature of the UTe_2 system, and unlike the superconductivity, is insensitive to the presence of crystalline disorder.

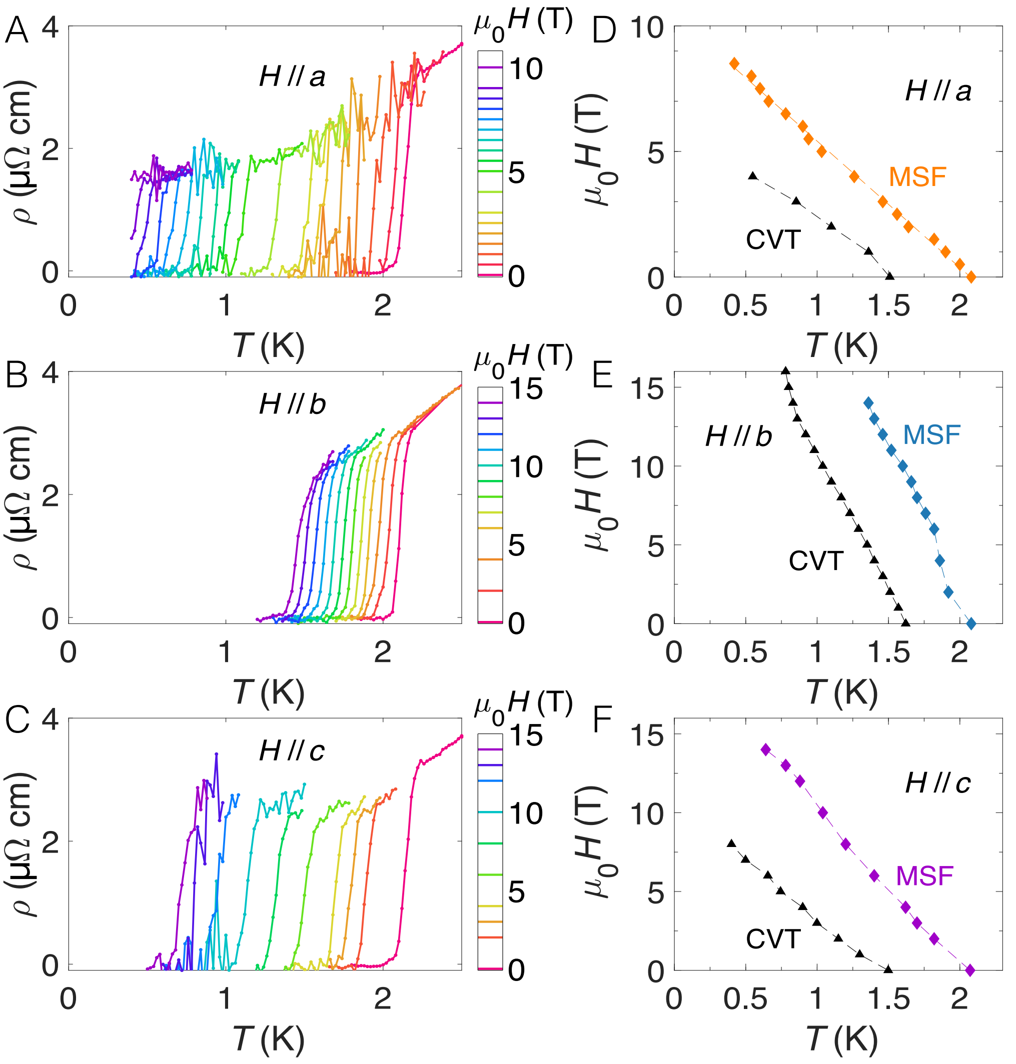

Fig. S4. Resistivity curves as a function of temperature for the RRR = 406 sample from Table 1 at intermediate magnetic fields with H applied along the (A) a-axis, (B) b-axis, and (D) c-axis. The strength of the applied field is indicated by the color scale. The corresponding profile of $T_c(H)$ is given in panels (D-F). A comparison is made between MSF-grown and CVT-grown UTe_2 using CVT data points from ref.[1]

Z. Wu, T. I. Weinberger, J. Chen, A. Cabala, D. V. Chichinadze, D. Shaffer, J. Pospisil, J. Prokleska, T. Haidamak, G. Bastien, V. Sechovsky, A. J. Hickey, M. J. Mancera-Ugarte, S. Benjamin, D. E. Graf, Y. Skourski, G. G. Lonzarich, M. Valiska, F. M. Grosche, A. G. Eaton

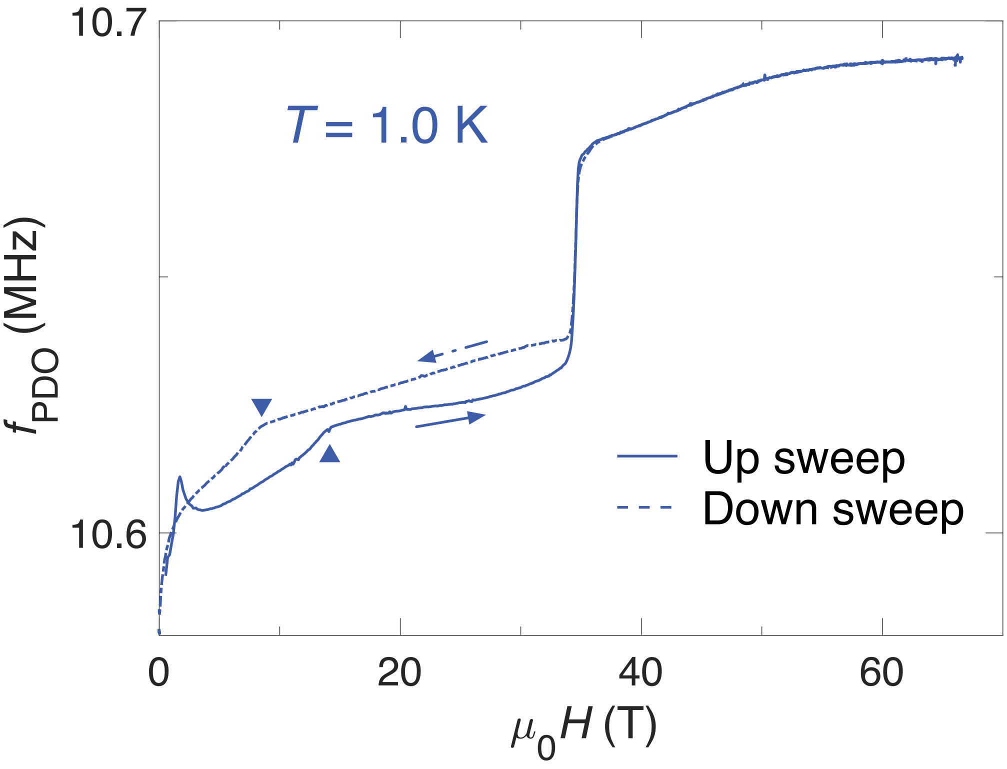

Fig. S5. Comparison of pulsed field PDO data, with $H \parallel b$ at 1.0 K, for the up-sweep (solid) and down-sweep (dashed) of a magnetic field pulse. Arrows indicate the direction of field sweep, with markers indicating the anomalous kink feature demarcating the transition from SC1 to SC2. We note that the sharp feature at the start of the up-sweep is likely due to a flux line moving in the SC1 state – similar features have been observed in prior pulsed field studies of superconductors.[2,3,4] The overlap of the rising and falling traces is very good above 35 T in the field polarised state, which is known to have minimal temperature dependence around 1 K, but is noticeably different below 35 T when superconductivity returns, where the temperature dependence is much more sensitive. This observation is consistent with heating effects from eddy currents and/or vortex motion during the pulse.

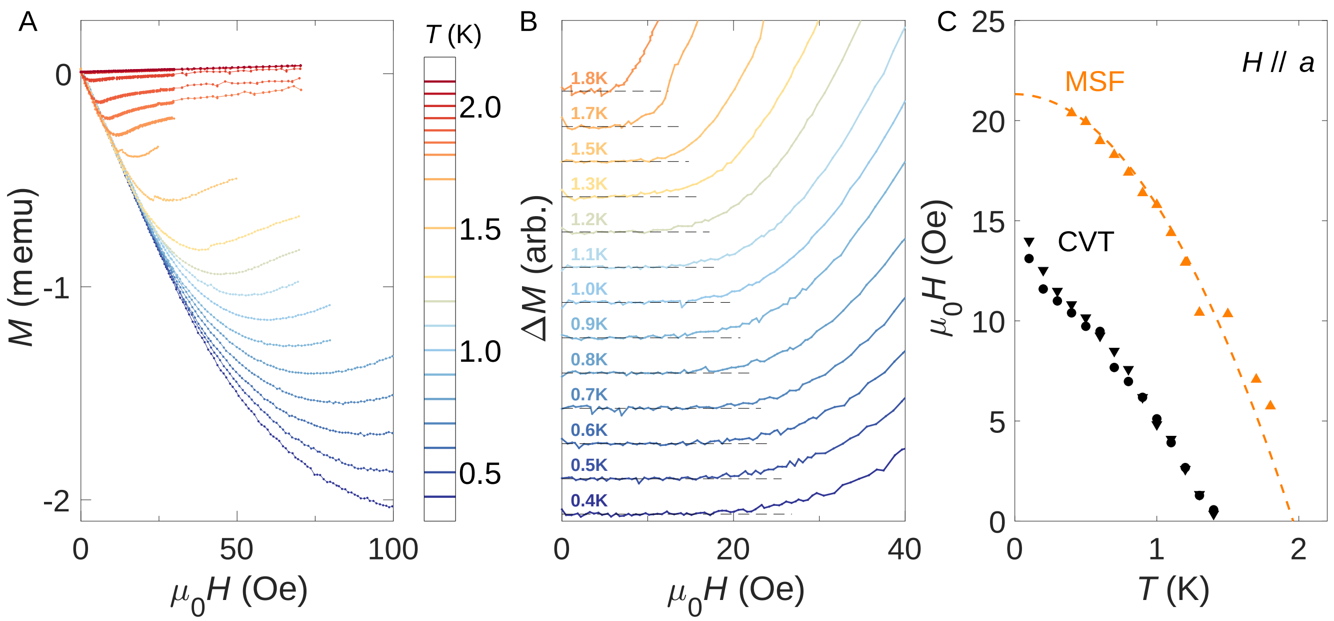

Fig. S6. Magnetization measurements at low temperatures and magnetic fields. Measurements were performed on the RRR = 105 sample from Table 1. (*A*) Isothermal measurements of the dc magnetic moment, *M*, as a function of magnetic field strength for $H \parallel a$. Temperature points are indicated by the color scale. (*B*) Magnetic moment versus field after subtracting a linear fit to the data for $\mu_0 H < 5$ Oe, as described in the text. Each curve is offset for clarity. (*C*) Magnetic field–temperature phase diagram of the Meissner state of UTe_2 for $H \parallel a$. A comparison of CVT data, from two separate samples marked by different symbols, is included from ref.[5]

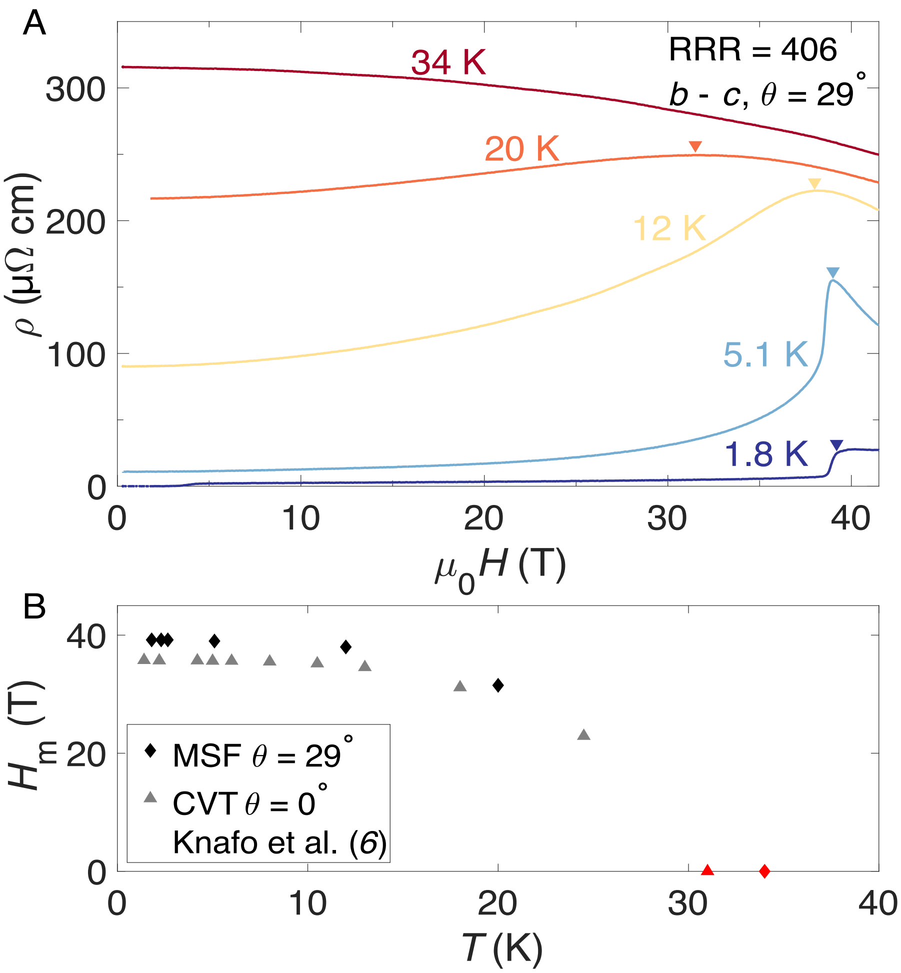

Fig. S7. (*A*) dc field resistivity data tracking the evolution of the metamagnetic transition at H_m (indicated with markers). (*B*) Comparison between the progression in temperature of H_m from panel (*A*) with that reported for a CVT sample in ref.[6] The red symbols indicate the first measured temperature point of each study at which the MM transition is no longer observed (which at elevated temperatures is identified as a broad maximum).

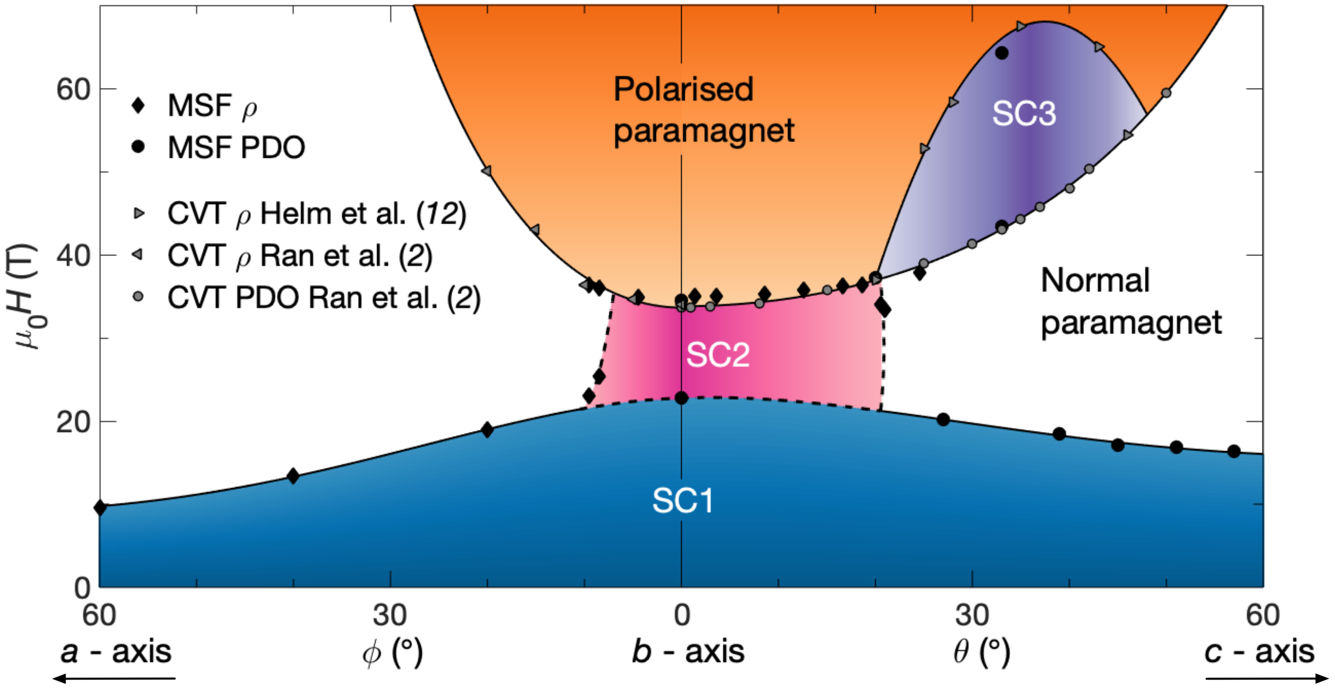

Fig. S8. Angular magnetic field phase diagram for MSF-grown UTe_2 for $\mu_0 H \leq 70$ T. Lines are as a guide to the eye. Comparative data points from pulsed field studies on CVT samples are from refs.[2,12]

Details of theoretical calculations

First-order transitions with narrow hysteresis. Low-energy fluctuations, which give rise to superconducting pairing of electrons, are known to appear in the vicinity of second-order phase transitions. There, the minimum of the free energy is shallow and the fluctuations are strong enough to cause the transition into a superconducting state. In contrast to second-order phase transitions, first-order phase transitions in general are not accompanied by divergent susceptibility and, therefore, usually do not promote pairing between itinerant electrons necessary for superconductivity to develop. Here we point out two special cases of first-order transitions where susceptibility can nevertheless be significantly enhanced. This enhancement of susceptibility can serve as the necessary 'glue' to create Cooper pairs.

The first case is a weak first order transition. Such a transition is characterized by a small jump of the order parameter that makes the transition behave almost as a continuous (second-order) one. In this case the two minima of the free energy are very close to each other, such that the susceptibility near the transition is enhanced. Weak first-order transitions are believed to cause superconductivity in various condensed matter systems.[14,15]

The second unusual case is a soft first-order transition with a narrow hysteresis due to low coercivity. Here the two minima are still far apart and the phase transition can be strong: the magnitude of the order parameter is not small as in a weak first-order transition case. However, the potential barrier between the two minima, which normally prohibits proliferation of fluctuations near first-order transitions, is shallow, see Fig. S9. This leads to the enhancement of fluctuations. In the limiting case of vanishing height of the potential barrier the system encounters a critical end point, where fluctuations are strong. Experimental evidence[16] points to the metamagnetic transition in UTe_2 to be a phase transition with narrow potential barrier. Therefore, it is natural to expect fluctuations near this transition to be present, and indeed to be strong enough to effect the pairing of electrons in the system.

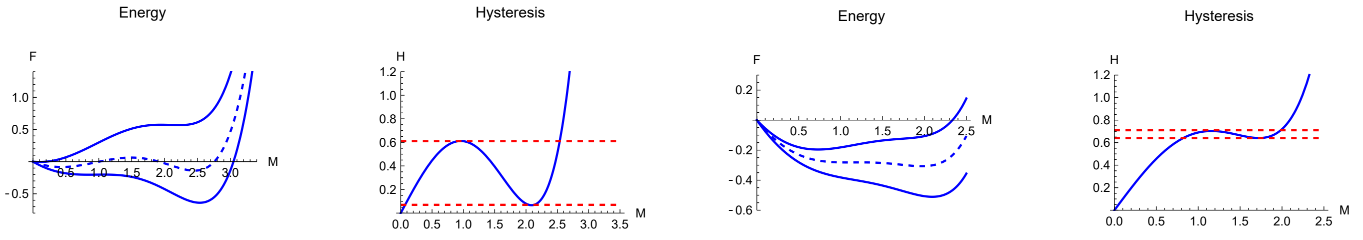

Fig. S9. An illustration of the correspondence between narrow hysteresis and the shallow potential barrier between the paramagnetic and metamagnetic minima of the free energy. Left: free energy as a function of the order parameter (magnetization) and magnetization curve for the case of wide hysteresis. One can see that the potential barrier (hump) between the two minima of the blue dashed curve is rather high; this suppresses fluctuations and the susceptibility is small. Right: narrow hysteresis leads to shallow barrier, hence, fluctuations are proliferating and the susceptibility is large even though the magnetization jump is not small.

Model for SC2. Here we present the details of the derivation of the spin-fluctuation interactions by integrating out the metamagnons. The action is given by

$$\mathcal{S} = -\sum_{n,\mathbf{q}} (i\Omega_n - \Omega_*(\mathbf{q}) + i\Gamma_m \mathrm{sgn}\Omega_n) m^\dagger_{\mathbf{q}} m_{\mathbf{q}} + \sum_{\mathbf{q}} \mu_e (m_{\mathbf{q}} + m^\dagger_{-\mathbf{q}}) M_* S_{\parallel} \qquad [2]$$

with $\Omega_*(\mathbf{q})$ given in Eq. 2 of the main text and where we use the Matsubara formalism; $\Omega_n = 2\pi nT$ are bosonic Matsubara frequencies. We also introduced the decay term Γ_m to account for the finite lifetime of the metamagnon. Here we assume that the metamagnon is a degree of freedom that stems from localized magnetic moments and not the itinerant fermionic degrees of freedom; this is consistent with recent theories of metamagnetic phase transitions in Kondo lattices.[17,18] This assumption allows us to integrate the bosons out in the standard way, which gives an effective dimensionless action for the fermions

$$\mathcal{S}_M[c, c^\dagger] = \beta \sum_{n,\mathbf{q}} J(i\Omega_n, \mathbf{q}) S_{\parallel}(i\Omega_n, \mathbf{q}) S_{\parallel}(-i\Omega_n, -\mathbf{q}), \qquad [3]$$

where

$$J(i\Omega_n, \mathbf{q}) = -\frac{\mu_e^2 M_*^2 \Omega_*(\mathbf{q})}{\Omega_*^2(\mathbf{q}) + \Omega_n^2 + \Gamma_m^2} \qquad [4]$$

are the effective ferromagnetic-fluctuation type interactions. This is equivalent to writing the interaction Hamiltonian as (using the four-momentum notation $q = (i\Omega_n, \mathbf{q})$, $p = (i\omega_m, \mathbf{p})$, etc)

$$\mathcal{H}_{int} = \sum_{q,k,p} J(q) c^\dagger_{k+q,s_1} \left(\boldsymbol{\sigma} \cdot \hat{\mathbf{M}}_*\right)_{s_1 s_2} c_{k,s_2} c^\dagger_{p-q,s_3} \left(\boldsymbol{\sigma} \cdot \hat{\mathbf{M}}_*\right)_{s_3 s_4} c_{p,s_4}. \qquad [5]$$

A proper treatment of the frequency dependence of the interaction would require solving the Eliashberg equations.[19,20,21] However, because close to the metamagnetic phase transition the interaction strength has a similar frequency dependence as in the case of phonons, i.e., the attraction happens mostly at low frequency, we can approximate $J(i\Omega_n, \mathbf{q}) \approx J(0, \mathbf{q})$. This gives us the form of the interactions as stated in the main text. We note that there are additional corrections due to the fact that the metamagnons, unlike phonons, are massive excitations away from the metamagnetic transition. This would likely modify the low energy cutoff of the theory in a more rigorous treatment, but the effect should be small close to the metamagnetic transition.

To get the gap equation and obtain the expression for T_c we first need to recast the interaction in the singlet/triplet pairing channels using the Pauli matrix completeness relation

$$2\delta_{s_1s_2}\delta_{s_3s_4} = \sum_{\mu=0,x,y,z} \sigma^{\mu}_{s_1s_3}\sigma^{\mu}_{s_2s_4} = \\ = \sum_{\mu}(\sigma^{\mu}i\sigma^{y})_{s_1s_3}\left[(\sigma^{\mu}i\sigma^{y})^{\dagger}\right]_{s_2s_4} \quad [6]$$

that yields

$$\mathcal{H}_{int} = \sum_{p,k} V_{\mu}(p;k) \\ \times \left(c^{\dagger}_{-ks_1}(\sigma^{\mu}i\sigma^{y})_{s_1s_3}c^{\dagger}_{ks_3}\right)\left(c_{ps_4}(\sigma^{\mu}i\sigma^{y})^{*}_{s_4s_2}c_{-ps_2}\right) \quad [7]$$

with $\mu = 0$ and $\mu = j = x, y, z$ corresponding to singlet and triplet pairing channels respectively, where

$$V_0(p;k) = -J^{(S)}_x(p-k) - J^{(S)}_y(p-k) - J^{(S)}_z(p-k) \\ V_x(p;k) = -J^{(A)}_x(p-k) + J^{(A)}_y(p-k) + J^{(A)}_z(p-k) \\ V_y(p;k) = J^{(A)}_x(p-k) - J^{(A)}_y(p-k) + J^{(A)}_z(p-k) \\ V_z(p;k) = J^{(A)}_x(p-k) + J^{(A)}_y(p-k) - J^{(A)}_z(p-k) \quad [8]$$

and where $J^{(S/A)}_j = J^{(S/A)}(q)\hat{M}_{*j}$ is proportional to the jth component of $\hat{\mathbf{M}}_*$, with

$$J^{(S/A)}(p-k) = \frac{J(p-k) \pm J(p+k)}{2}. \quad [9]$$

The functions $J^{(S/A)}(p-k)$ can be decomposed into terms transforming according to particular irreducible representations of the crystalline point group symmetries. To leading order, this can be achieved by expanding $J^{(S/A)}(p-k)$ in momentum and keeping only the leading term. This yields

$$J^{(S)}(p-k) \sim -\frac{\mu_e^2 M_*^2 \Omega_*(0)}{\Omega_*^2(0) + \Gamma_m^2}, \\ J^{(A)}(p-k) \sim -\frac{2\mu_e^2 M_*^2 \Omega_*^2(0)}{(\Omega_*^2(0) + \Gamma_m^2)^2}\sum_j \kappa_j p_j k_j. \quad [10]$$

We next introduce the gap functions via a Hubbard-Stratonovich transformation:

$$H_{\Delta} = \sum \Delta^{(\mu)}(p)(\sigma^{\mu}i\sigma^{y})_{s_1s_2}c^{\dagger}_{ps_1}c^{\dagger}_{-ps_2}. \quad [11]$$

The corresponding linearized gap equation (valid in the weak coupling approximation) reads

$$\Delta^{(\mu)}(p) = -T\sum_k V_{\mu}(p;k)\Pi_{\mu\mu'}(k)\Delta^{(\mu')}(k) \quad [12]$$

where

$$\Pi_{\mu\mu'}(k) = \mathrm{Tr}\left[\sigma^{\mu}i\sigma^{y}G(k)(\sigma^{\mu'}i\sigma^{y})^{*}G^{T}(-k)\right] \quad [13]$$

is the particle-particle bubble (before the Matsubara sum), the trace is over spin indices, and

$$G(k) = \frac{1}{i\omega_n - \varepsilon(\mathbf{k}) - \mu_e\mathbf{H}\cdot\boldsymbol{\sigma}} = \\ = \frac{i\omega_n - \varepsilon(\mathbf{k}) + \mu_e\mathbf{H}\cdot\boldsymbol{\sigma}}{(i\omega_n - \varepsilon(\mathbf{k}))^2 - \mu_e^2H^2} \quad [14]$$

is the Green's function that includes the Zeeman term.

For the special case of $\mathbf{H}$ along the y axis, since we can assume that the Fermi surfaces are spin polarized close to the phase transition, we take $G(k) \propto \frac{1}{2}(\pm 1 + \sigma^{y})$ (with $\pm$ corresponding to the two spin split Fermi surfaces). One can then check that the $\mu = 0, y$ channels vanish while

$$\Pi_{xx}(k) = \Pi_{zz}(k) = \frac{1}{\omega_n^2 - (\varepsilon(\mathbf{k}) \pm \mu_e H)^2} \\ \Pi_{xz}(k) = \pm\Pi_{zx}(k) = \frac{i}{\omega_n^2 - (\varepsilon(\mathbf{k}) \pm \mu_e H)^2} \quad [15]$$

with the ± in the denominator corresponding to the Fermi surface with spin aligned against and with the magnetic field, respectively. Note that the relevant interactions are thus $V_x = V_z = J_y^{(A)}$, so that the $\mathbf{d} = d(1, 0, \pm i)$ vector is interestingly non-unitary, similar to that proposed in.[22]

Combining with our knowledge of the $\mathbf{d}$-vector, we obtain the final equation for Δ with $\Delta^{(x)}(\mathbf{k}) = -i\Delta^{(z)}(\mathbf{k}) = \Delta(\mathbf{k})$ (summing over both spin polarized Fermi surfaces for additional factor of two, another factor of two from the eigenvalue of Π matrix, and neglecting form factors from Fermi surface shapes):

$$\Delta(\mathbf{p}) = \frac{8\nu\mu_e^2 M_*^2 \Omega_*^2(0)}{(\Omega_*^2(0) + \Gamma_m^2)^2} \log\frac{1.13\Lambda}{T_c} \sum_j \kappa_j \int p_j k_j \Delta(\mathbf{k}) dS_{FS} \qquad [16]$$

where the surface integral is taken over the Fermi surface. The solutions are thus $\Delta(\mathbf{p}) \propto p_j$, with different j belonging to different irreps. Possibilities include the $B_{1u} + iB_{3u}$ irrep combination of D_{2h} irrep for $j = y$ (corresponding to the B_u irrep of C_{2h} in the presence of a magnetic field along the b axis) as proposed in[22] ; or, for either $j = x$ or $j = z$, the $A_u + iB_{2u}$ combination (A_u irrep of C_{2h}) that was considered in.[23] Regardless of the form of the order parameter, within weak coupling we obtain an expression for T_c in Eq. 3 of the main text with a parameter κ that accounts for any form factors resulting from the integration over the Fermi surface. As the form of the order parameter is still under debate, we simply consider κ as a phenomenological parameter.

Model for SC1. To model SC1, let us assume that the FM or AFM fluctuation-induced interaction at zero external field has the form

$$H_{int} = V \sum_{k,p} \left(c^\dagger_{-ks_1}(\hat{\mathbf{d}}(\mathbf{k}) \cdot \boldsymbol{\sigma} i\sigma^y)_{s_1 s_3} c^\dagger_{ks_3}\right) \left(c_{ps_4}(\hat{\mathbf{d}}(\mathbf{p}) \cdot \boldsymbol{\sigma} i\sigma^y)^*_{s_4 s_2} c_{-ps_2}\right) \qquad [17]$$

where V is a constant. Unlike the SC2 model, here we assume that V is independent of the applied field and arises from intrinsic spin fluctuations present in the ground state of the system in the absence of any field. It is then easy to see that the self-consistent gap functions have the form $\Delta(\mathbf{p}) = \mathbf{d}(\mathbf{k}) \cdot \boldsymbol{\sigma} i\sigma^y$. For simplicity, let us quantize spin along the direction of $\mathbf{H}$, so that

$$G(k) = \begin{pmatrix} \frac{1}{i\omega_n - \varepsilon(\mathbf{k}) - h + i\Gamma_e \mathrm{sgn}\omega_n} & 0 \\ 0 & \frac{1}{i\omega_n - \varepsilon(\mathbf{k}) + h + i\Gamma_e \mathrm{sgn}\omega_n} \end{pmatrix} \equiv \begin{pmatrix} G_\uparrow(k) & 0 \\ 0 & G_\downarrow(k) \end{pmatrix} \qquad [18]$$

where $h = \mu_e H = \mu_B g_e H/2$ and we introduced the electron decay rate Γ_e to account for disorder. Evaluating the trace we then obtain the following self-consistency gap equation (c.f.[24,25]):

$$\begin{aligned} 1 = -VT \sum_k \Big[&(|d_x(\mathbf{k})|^2 + |d_y(\mathbf{k})|^2)(G_\uparrow(k)G_\uparrow(-k) + G_\downarrow(k)G_\downarrow(-k)) \\ &+ |d_z(\mathbf{k})|^2 (G_\uparrow(k)G_\downarrow(-k) + G_\uparrow(-k)G_\downarrow(k)) \Big]. \end{aligned} \qquad [19]$$

We can generalize to any orientation of the magnetic field by using a coordinate-free notation:

$$\begin{aligned} 1 = -VT \sum_k \Big[&|d_\perp(\mathbf{k})|^2 (G_\uparrow(k)G_\uparrow(-k) + G_\downarrow(k)G_\downarrow(-k)) \\ &+ |d_\parallel(\mathbf{k})|^2 (G_\uparrow(k)G_\downarrow(-k) + G_\uparrow(-k)G_\downarrow(k)) \Big]. \end{aligned} \qquad [20]$$

The Matsubara sums for the $d_\perp$ part are the same as without magnetic field (these components are thus insensitive to the magnetic field), and in the absence of disorder we obtain the usual logarithmic term. With disorder, we get

$$\sum_n \int d\varepsilon G_\uparrow(k)G_\uparrow(-k) = \sum_n \int d\varepsilon G_\downarrow(k)G_\downarrow(-k) = \log\frac{1.13\Lambda}{T} - \psi\left(\frac{1}{2} + \frac{\Gamma_e}{2\pi T}\right) + \psi\left(\frac{1}{2}\right). \qquad [21]$$

Evaluating the sum for the $d_\parallel$ term, on the other hand, gives (assuming $h \ll \Lambda$)

$$\sum_n \int d\varepsilon G_\uparrow(k)G_\downarrow(-k) = \log\frac{1.13\Lambda}{T} - \mathrm{Re}\left[\psi\left(\frac{1}{2} + \frac{\Gamma_e + ih}{2\pi T}\right) - \psi\left(\frac{1}{2}\right)\right] \qquad [22]$$

where ψ is the digamma function. After doing the sum over k in Eq. (20), we then have

$$1 = -\tilde{V}\left[\log\frac{1.13\Lambda}{T} - (1 - c(\theta,\phi))\left[\psi\left(\frac{1}{2} + \frac{\Gamma_e}{2\pi T}\right) - \psi\left(\frac{1}{2}\right)\right] - c(\theta,\phi)\mathrm{Re}\left[\psi\left(\frac{1}{2} + \frac{\Gamma_e + ih}{2\pi T}\right) - \psi\left(\frac{1}{2}\right)\right]\right]$$

with

$$\tilde{V} = 2V\nu \int |\mathbf{d}(\mathbf{k})|^2 dS_{FS}$$

where ν is the density of states and

$$0 < c(\theta,\phi) = \frac{\int |d_\parallel(\mathbf{k})|^2 dS_{FS}}{\int |\mathbf{d}(\mathbf{k})|^2 dS_{FS}} < 1$$

is a form factor that we can treat as a phenomenological parameter that only depends on the direction of the field **H**. This is most conveniently re-written as

$$\log\frac{T_c(\mathbf{H},\Gamma_e)}{T_{c0}} = -(1-c(\theta,\phi))F\left(\frac{\Gamma_e}{T_c}\right) - c(\theta,\phi)F\left(\frac{\Gamma_e+ih}{T_c}\right)$$

where $F(x) = \mathrm{Re}\left[\psi\left(\frac{1}{2}+\frac{x}{2\pi}\right)-\psi\left(\frac{1}{2}\right)\right]$, leading to the expression

$$\log\frac{T_c^{(SC1)}}{T_{c0}^{(SC1)}} = -(1-c(\theta,\phi))F\left(\frac{\Gamma_e}{T_c}\right) - c(\theta,\phi)F\left(\frac{\Gamma_e+ih}{T_c}\right) \qquad [23]$$

that was used to obtain the plots in Fig. 7. We used the parameters of $T_c = 2.1$ K, $\mu_e = 0.2\mu_{\mathrm{B}}$ in panel *A*, and $c = 0.7 + 0.1\sin^2\theta$, $T = 0.35$ K (0.035 K) and $\Gamma_e = 0$ (0.2) to model the clean (dirty) sample in panel *B*. (We neglect the anisotropy of H_c seen in experiment in this case.)

References

1. S Ran, et al., Nearly ferromagnetic spin-triplet superconductivity. *Science* **365**, 684–687 (2019).
2. S Ran, et al., Extreme magnetic field-boosted superconductivity. *Nat. Phys.* **15**, 1250–1254 (2019).
3. M Nikolo, et al., Upper critical and irreversibility fields in $Ba(Fe_{0.95}Ni_{0.05})_2As_2$ and $Ba(Fe_{0.94}Ni_{0.06})_2As_2$ pnictide bulk superconductors. *J. Supercond.* **30**, 331–341 (2017).
4. MP Smylie, et al., Anisotropic upper critical field of pristine and proton-irradiated single crystals of the magnetically ordered superconductor $RbEuFe_4As_4$. *Phys. Rev. B* **100**, 054507 (2019).
5. C Paulsen, et al., Anomalous anisotropy of the lower critical field and Meissner effect in UTe_2. *Phys. Rev. B* **103**, L180501 (2021).
6. W Knafo, et al., Magnetic-field-induced phenomena in the paramagnetic superconductor UTe_2. *J. Phys. Soc. Jpn.* **88**, 063705 (2019).
7. DR Tilley, J Tilley, *Superfluidity and superconductivity*. (Routledge, Oxfordshire, UK), (1990).
8. M Konczykowski, LI Burlachkov, Y Yeshurun, F Holtzberg, Evidence for surface barriers and their effect on irreversibility and lower-critical-field measurements in Y-Ba-Cu-O crystals. *Phys. Rev. B* **43**, 13707–13710 (1991).
9. M Abdel-Hafiez, et al., Temperature dependence of lower critical field $H_{c1}(T)$ shows nodeless superconductivity in FeSe. *Phys. Rev. B* **88**, 174512 (2013).
10. RA Klemm, JR Clem, Lower critical field of an anisotropic type-II superconductor. *Phys. Rev. B* **21**, 1868–1875 (1980).
11. K Ishihara, et al., Anisotropic enhancement of lower critical field in ultraclean crystals of spin-triplet superconductor candidate UTe_2. *Phys. Rev. Res.* **5**, L022002 (2023).
12. T Helm, et al., Field-induced compensation of magnetic exchange as the possible origin of reentrant superconductivity in UTe_2. *Nat. Commun.* **15**, 37 (2024).
13. CE Frank, et al., Orphan High Field Superconductivity in Non-Superconducting Uranium Ditelluride (2023).
14. D Hu, et al., Structural and Magnetic Phase Transitions near Optimal Superconductivity in $BaFe_2(As_{1-x}P_x)_2$. *Phys. Rev. Lett.* **114**, 157002 (2015).
15. H Li, et al., Observation of Unconventional Charge Density Wave without Acoustic Phonon Anomaly in Kagome Superconductors AV_3Sb_5 (A = Rb, Cs). *Phys. Rev. X* **11**, 031050 (2021).
16. Reinforcement of superconductivity by quantum critical fluctuations of metamagnetism in UTe_2, author = Tokiwa, Y. and Opletal, P. and Sakai, H. and Kambe, S. and Yamamoto, E. and Kimata, M. and Awaji, S. and Sasaki, T. and Aoki, D. and Haga, Y. and Tokunaga, Y. *Phys. Rev. B* **109**, L140502 (2024).
17. BH Bernhard, Metamagnetism and tricritical behavior in the Kondo lattice model. *Phys. Rev. B* **106**, 054436 (2022).
18. C Thomas, S Burdin, C Lacroix, Metamagnetic transition in the two f orbitals Kondo lattice model (2023).
19. F Marsiglio, Eliashberg theory: A short review. *Ann. Phys.* **417**, 168102 (2020).
20. A Abanov, AV Chubukov, J Schmalian, Quantum-critical theory of the spin-fermion model and its application to cuprates: Normal state analysis. *Adv. Phys.* **52**, 119–218 (2003).
21. AV Chubukov, A Abanov, I Esterlis, SA Kivelson, Eliashberg theory of phonon-mediated superconductivity — When it is valid and how it breaks down. *Ann. Phys.* **417**, 168190 (2020).
22. AH Nevidomskyy, Stability of a Nonunitary Triplet Pairing on the Border of Magnetism in UTe_2 (2020).
23. JJ Yu, Y Yu, DF Agterberg, S Raghu, Theory of the low- and high-field superconducting phases of UTe_2. *Phys. Rev. B* **107**, 214510 (2023).
24. PA Frigeri, DF Agterberg, A Koga, M Sigrist, Superconductivity without Inversion Symmetry: MnSi versus $CePt_3Si$. *Phys. Rev. Lett.* **92**, 097001 (2004).
25. V Mineev, Upper critical field in ferromagnetic metals with triplet pairing. *Ann. Phys.* **417**, 168139 (2020).
